# *ELN* missense variant in patient with mid-aortic syndrome case report

**DOI:** 10.1186/s12872-022-02965-3

**Published:** 2022-12-03

**Authors:** Thao Nguyen Phan, Long Hoang Luong, The Huy Nguyen, Hoang Duc Nguyen, Tran Thuy Nguyen

**Affiliations:** 1 National E Hospital, Hanoi, Vietnam; 2 VNU University of Medicine and Pharmacy, 144 Xuan Thuy Street, Cau Giay, Hanoi, Vietnam

**Keywords:** Mid-aortic syndrome, *ELN* gene, *ELN* variant, Klinefelter syndrome, Loss-of-function mutation, Abdominal aorta, Stenosis, Stroke, Coarctation, Elastin

## Abstract

**Background:**

Mid-aortic syndrome (MAS) is characterized by the congenital coarctation of the abdominal aorta, abdominal and limb claudication, and hypertension. The etiology of this disorder is very diverse and often manifests in conjunction with Takayasu's arteritis, Williams-Beurens syndrome, and neurofibromatosis. The isolated mid-aortic syndrome is very rare with only a few cases reported in the literature.

**Case presentation:**

A 45 years old man was admitted to the Emergency Department with sudden muscle weakness and facial paralysis on the left side. Imaging studies reveal right middle cerebral artery infarction at the M1 section. Incidental findings include multiple moderate to severe stenoses in the right internal carotid artery, and total abdominal aorta occlusion. A variant at the *ELN* gene (*Elastin*, OMIM*130,160): c.1768G > A/wt (p.Ala590Thr) was identified.

**Conclusion:**

This is the first reported case of *ELN* related mid-aortic syndrome in Vietnam which was diagnosed through careful clinical and genetic workup. The finding of mid-aortic syndrome, in this case, was incidental and the decision to reverse the occlusion was postponed as there was no immediate risk of renal failure or reduced blood flow to the lower limb.

## Background

Mid-aortic syndrome (MAS) is distinguished by the congenital coarctation of the abdominal aorta, abdominal and limb claudication, and hypertension [1]. The etiology of the disease is either due to genetic defects (Williams-Beurens syndrome, pathogenic variants at *NF1, JAG1, ELN* gene…), autoimmune disorder (Takayasu's arteritis), or idiopathic [2]. MAS often occur in conjunction with other vascular diseases, most notably cerebrovascular diseases with incidence up to 44%[3]. The disorder is extremely rare, with the prevalence of less than 1/1,000,000, and patient life expectancy varies depending on the etiology [4, 5]. Here we report a case admitted to the Emergency Department (ER) with stroke following a diagnosis of mid-aortic syndrome. Complete genetic workup was conducted which revealed the patient had XXY aneuploidy in conjunction with a missense variant at *ELN*, which was likely pathogenic and attributable for this patient’s condition.

## Case presentation

A 45 years old male was admitted to the ER with sudden muscle weakness and facial paralysis on the left side. The patient’s history consists of intermittent claudication without any treatment. The patient’s vitals show no abnormalities except for high blood pressure with 159/80 mmHg. The patient’s brain CT scan shows acute infarction in the right cerebral hemisphere suggesting right middle cerebral artery infarction. Computed tomography angiography shows severe stenosis of the right internal carotid artery, infarction of the M1 section of the right middle cerebral artery. Cardiosonography reveals mild mitral stenosis, moderate tricuspid regurgitation 2/4, and mild pulmonary artery hypertension. Carotid artery Doppler ultrasound remarks a large atherosclerotic plaque in the right carotid bifurcation and right internal carotid artery that block more than 50% of the right carotid artery. Carotid artery Doppler ultrasound also reveals left subclavian steal syndrome and multiple stenoses as well as atherosclerosis in common carotid arteries, left internal carotid artery, external carotid artery, right vertebral artery, and right subclavian artery. Incidentally, abdominal aorta Doppler ultrasound found a chronic abdominal aortic occlusion at the location from left renal artery to bilateral external iliac arteries. Lower extremities Doppler ultrasound shows normal circulation received mostly from collateral circulation from deep femoral arteries. However, as the priority at the time was to resolve the patient’s cerebral artery stroke, and no immediate renal failure or reduced blood flow to the lower limb was noted, we decided to delay any work-up regarding the abdominal aorta.

Emergency management for this patient was to re-establishment of the cerebral circulation. The patient at the time of hospitalization met the requirements for using of tissue plasminogen activator. The patient was administered Alteplase with the dose of 0.9 mg/kg and transferred to Interventional Cardiovascular Unit for mechanical thrombectomy with a Solitaire device under the guide of digital subtraction angiography (DSA). Post-intervention, the patient achieved a score of 3 using modified treatment in cerebral ischemia (mTICI) score.

The patient was transferred to the Department of Cardiology on the following day. An angiogram of the patient’s abdominal aorta was taken using sheath 8F with right brachial artery insertion. The angiogram shows complete occlusion of the abdominal aorta at the level of 10 mm above aortic bifurcation, with no abnormalities in internal and external iliac arteries, except for 50% narrowing in the left external iliac artery and occlusion in the distal section of the left internal iliac artery. As the patient presented with no apparent renal insufficiency or lower limb ischemia, after weighing in the risk and benefit, the decision to reverse the mid-aortic occlusion was postponed indefinitely and the patient were scheduled for regular check-up.

The patient was discharged 1 week later in good condition and with full recovery of limb mobility and no apparent sequelae of stroke was noted upon subsequent visit.

### Genetic workup

Blood samples were collected for genetic workup. In this patient, karyotyping and CNV analysis further revealed XXY aneuploidy, the patient had no children and was suspected of low fertility due to Klinefelter syndrome. However, the XXY aneuploidy has not been attributable to mid-aortic syndrome therefore we suspected a monogenic cause. Whole Exome sequencing (GeneSolution, Ho Chi Minh City, Vietnam) was conducted for this patient, data analysis focused on genes related to cardiovascular diseases and connective tissue disorders. The result revealed a variant at the *ELN* gene (*Elastin*, OMIM*130,160): c.1768G > A/wt (p.Ala590Thr). To assess the pathogenicity and any previously reported pathogenic variant of *ELN*, we used Human Gene Mutation Database, LOVD, ClinVar as reference databases and followed American College of Medical Genetics (ACMG) Guidelines for interpretation of pathogenic variant [6]. The variant was observed at a very low frequency in the general population and the Asian population at a frequency of 0.000012 (Genome Aggregation Database March 6, 2019, v2.1.1), this variant was not previously reported in patients with Mid-aortic syndrome, and the ACMG verdict was “Likely Pathogenic”.

## Discussion and conclusions

The likely cause of MAS, in this case, would be the variant at the *ELN* gene, as the *ELN* loss-of-function (deletion of 1.5 Mb on chromosome 7q11.23) has been linked to aortic stenosis and connective tissue abnormality in Williams-Beurens syndrome. *ELN* encodes for a protein called tropoelastin, which later forms Elastin, a major component of elastic fiber that provides structural integrity and flexibility to connective tissue. There are only a few cases in the literature that reported isolated MAS with *ELN* pathogenic variant being the only genetic defect [7], as *ELN* have been classically linked to other vascular disorder such as supravalvular aortic stenosis or pulmonary artery stenosis. In cases where *ELN* pathogenic variant was found, the stenosis was reported to happen at the perirenal level (from below the renal arteries to above the celiac).

In this patient we found a variant at the *ELN* gene (c.1768G > A, p.Ala590Thr), which was concluded to be causative of this patient’s condition based on the ACMG Guidelines for interpretation of pathogenic variants. However, further in vitro and in vivo analyses need to be conducted to confirm the pathogenicity of this *ELN* variant. The XXY aneuploidy (Klinefelter syndrome) would likely cause infertility in this patient and potentially other heart defects, however, upon further inspection, no congenital heart disease was observed. Klinefelter syndrome has not been reported to cause mid-aortic syndrome as there has only been one report of Klinefelter syndrome with mild aortic stenosis with pulmonary stenosis [8].

**Fig. 1:**
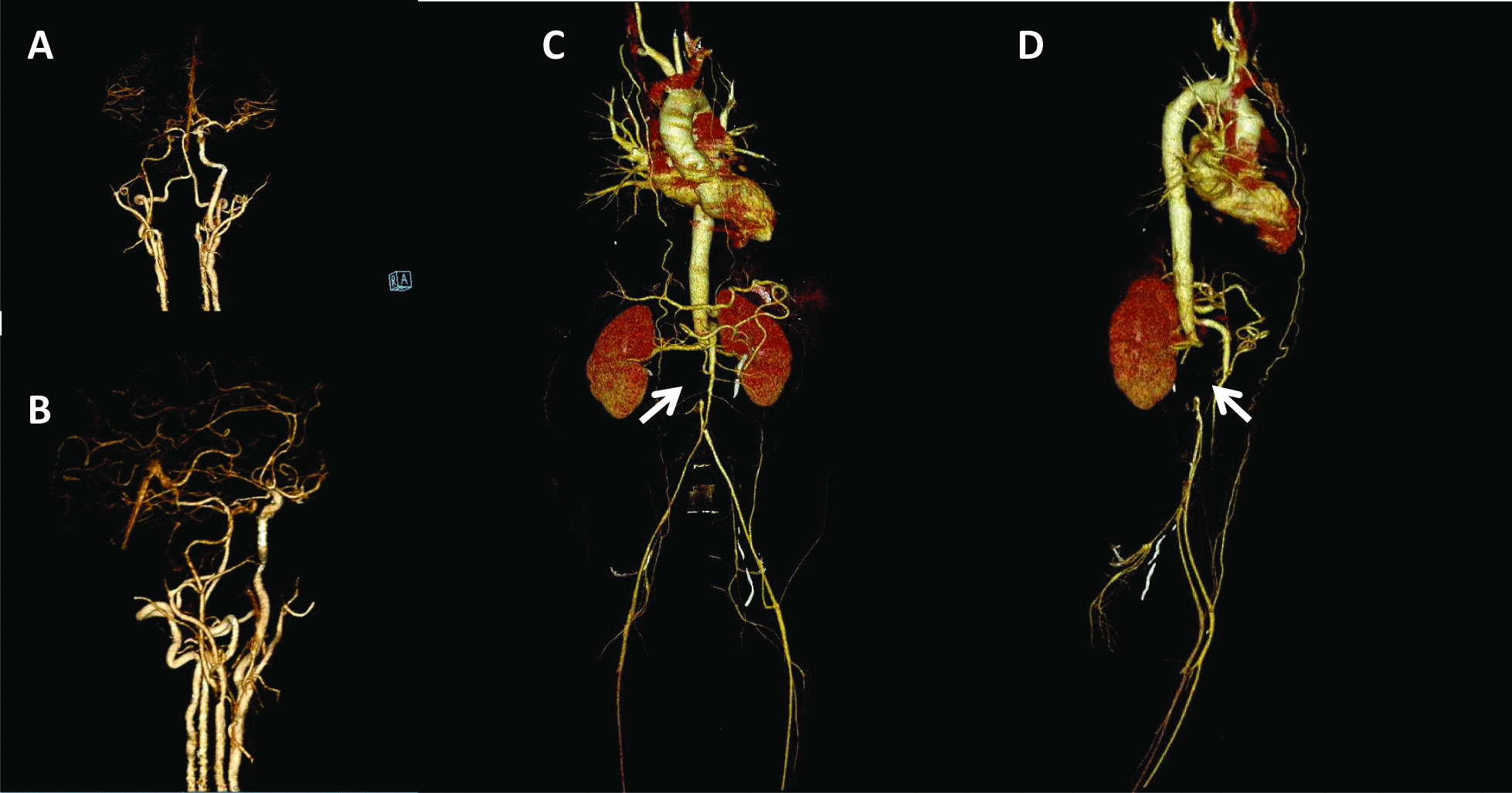
Pre-intervention computed tomography (CT) angiography of the patient’s stenosis of cerebral artery (**A**), carotid artery (**B**); CT angiography of abdominal and renal artery (**C**, **D**) complete occlusion of the abdominal aorta at the level of 10 mm above aortic bifurcation (Arrows)

This is the first reported case of *ELN* related mid-aortic syndrome in Vietnam which was diagnosed through careful clinical and genetic workup. The finding of mid-aortic syndrome, in this case, was incidental and the decision to reverse the occlusion was postponed as no immediate renal failure or reduced blood flow to the lower limb was noted. The collateral circulation of this patient was enough to maintain lower limb blood supply even with full occlusion of the abdominal aorta, therefor leading to the late diagnosis of MAS in this case (Fig. 1).

## Data Availability

The authors declare that the data supporting the findings of this study are available within the article.

## Abbreviations

MAS: Mid-aortic syndrome
ER: Emergency room/emergency department
ACMG: American college of medical genetics
DSA: Digital subtraction angiography
mTICI: Modified treatment in cerebral ischemia
CNV: Copy number variation
